# Response to PD‐1 blockade in a patient with mucosal melanoma of the middle ear: Case report

**DOI:** 10.1002/ccr3.3424

**Published:** 2020-10-29

**Authors:** Hiroki Komatsuda, Takumi Kumai, Seigo Ueda, Yui Hirata‐Nozaki, Tatsuya Hayashi, Yasuaki Harabuchi

**Affiliations:** ^1^ Department of Otolaryngology‐Head & Neck Surgery Asahikawa Medical University Asahikawa Japan; ^2^ Department of Innovative Head & Neck Cancer Research and Treatment (IHNCRT) Asahikawa Medical University Asahikawa Japan; ^3^ Wakkanai E.N.T Clinic Wakkanai Japan

**Keywords:** middle ear, PD‐1 blockade, primary mucosal melanoma

## Abstract

PD‐1 blockade is a feasible approach in treating mucosal malignant melanomas of the middle ear.

## Graphical abstract

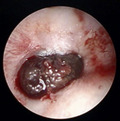

## 1 INTRODUCTION

Primary mucosal malignant melanomas of the middle ear are rare, described in only a few case reports worldwide. We report a case of primary mucosal malignant melanoma of the middle ear treated with a PD‐1 blockade. We present the case of an 80‐year‐old woman complaining of hearing difficulty in the right ear. A tumor extended from the right eardrum to the external auditory canal. A tumor biopsy revealed mucosal malignant melanoma. The patient was treated with nivolumab. The patient remained symptom‐free for 60 months. PD‐1 blockade is a feasible approach in treating mucosal malignant melanomas of the middle ear.

Primary mucosal malignant melanomas of the head and neck are relatively rare with most primary lesions generally found in the nasal cavity, paranasal sinuses, oral cavity, and pharyngeal mucosa. Primary mucosal malignant melanomas of the middle ear are extremely rare, described in only a few case reports worldwide.^1^, ^2^, ^3^, ^4^, ^5^, ^6^, ^7^, ^8^, ^9^, ^10^, ^11^, ^12^ Since the outcomes of current treatment options for mucosal melanomas are poor, a novel approach is required to treat mucosal malignant melanomas of the middle ear. Immune checkpoint inhibitors have recently been reported to be effective in treating many types of cancer, especially cutaneous melanoma.^13^ The efficacy of immune checkpoint inhibitors for the treatment of mucosal melanomas, however, has not been fully evaluated. We report a case of primary mucosal malignant melanoma of the middle ear that was effectively treated with nivolumab, an anti‐PD‐1 antibody.

## 2 CASE REPORT

**FIGURE 1:**
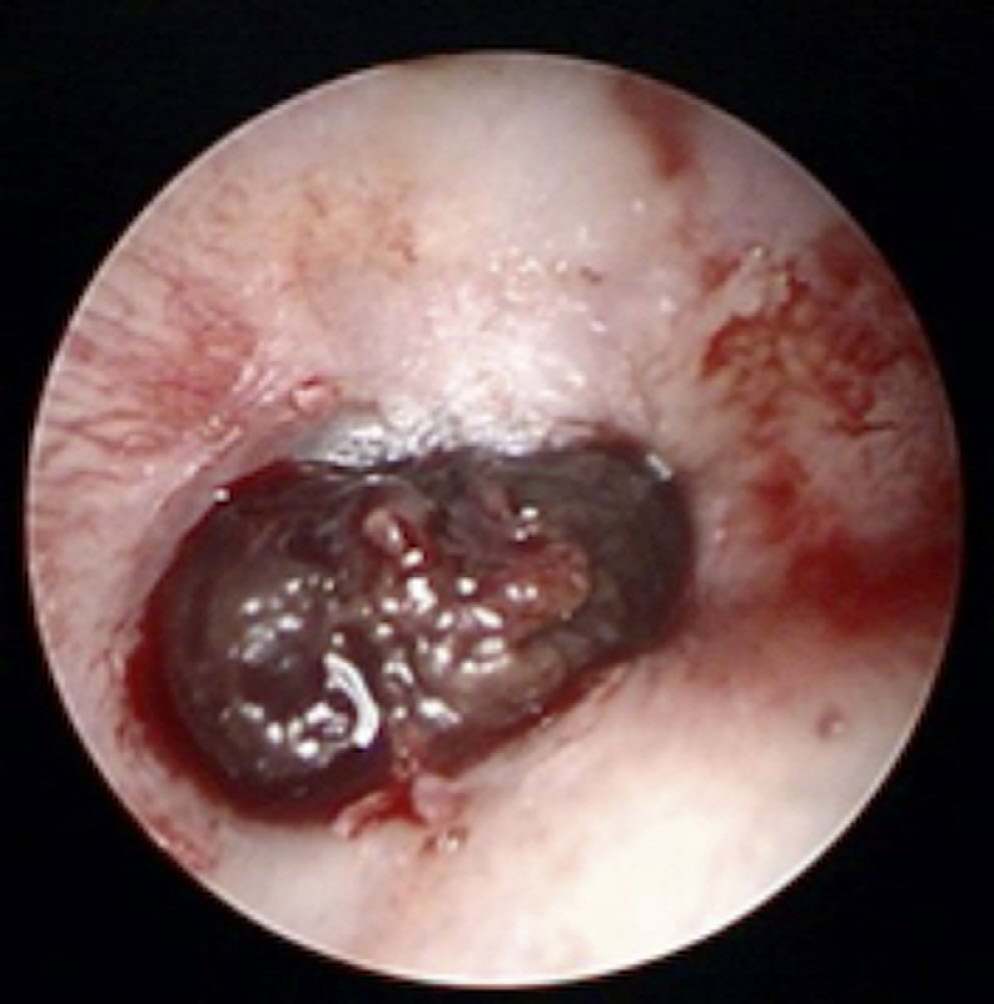
The tumor image before the treatment. A dark‐red elevated lesion with bleeding developed beyond the eardrum to the external auditory canal

**FIGURE 2:**
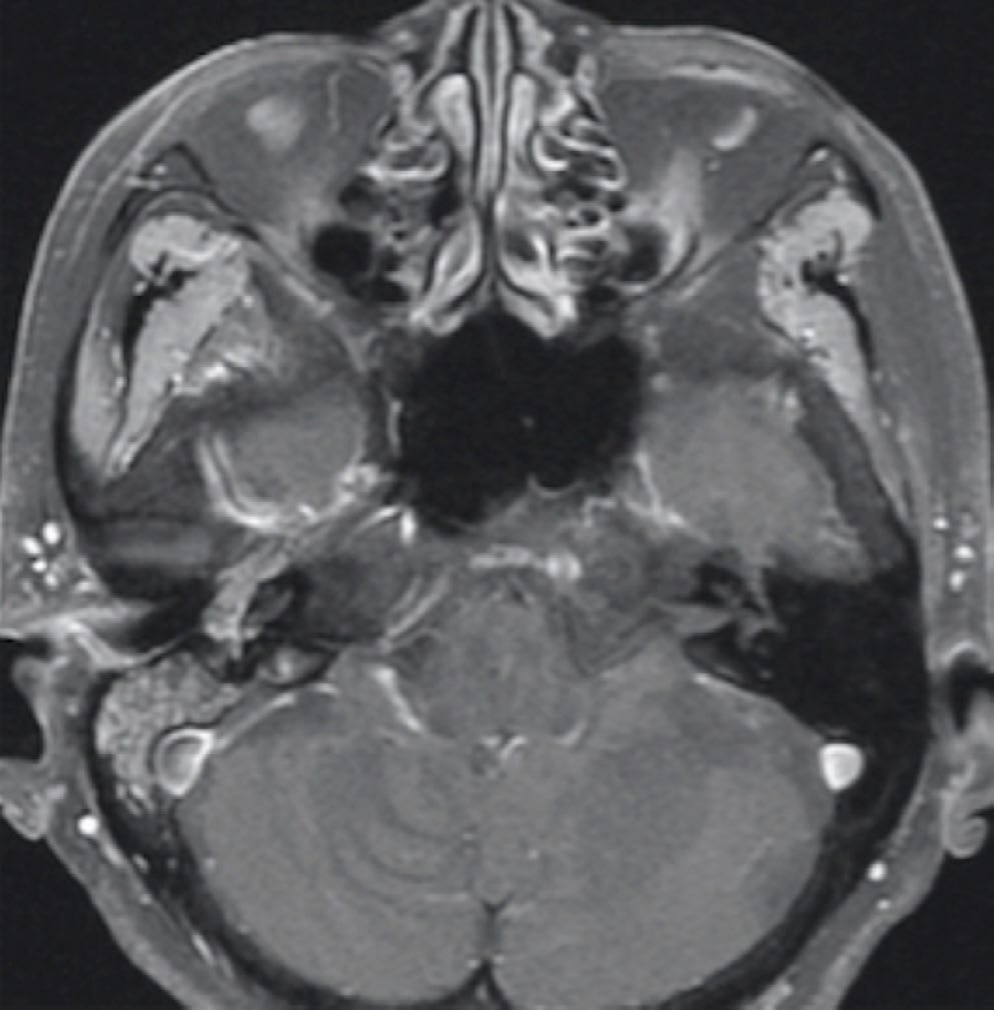
T1‐weighted axial MRI showed the tumor developed from the middle ear cavity to the external auditory canal

**FIGURE 3:**
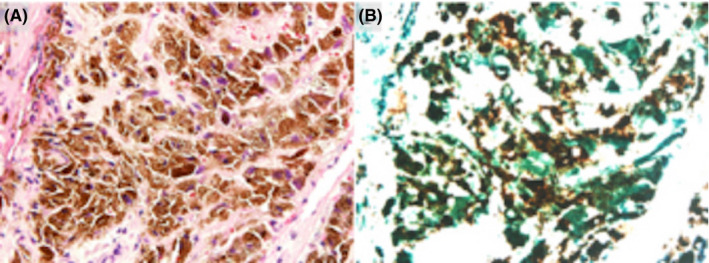
The pathological finding of tumor. A, Hematoxylin and eosin staining, original magnification ×200. B, The tumor cells ware positive for PD‐L1. (PD‐L1 SP142 staining, original magnification ×200)

**FIGURE 4:**
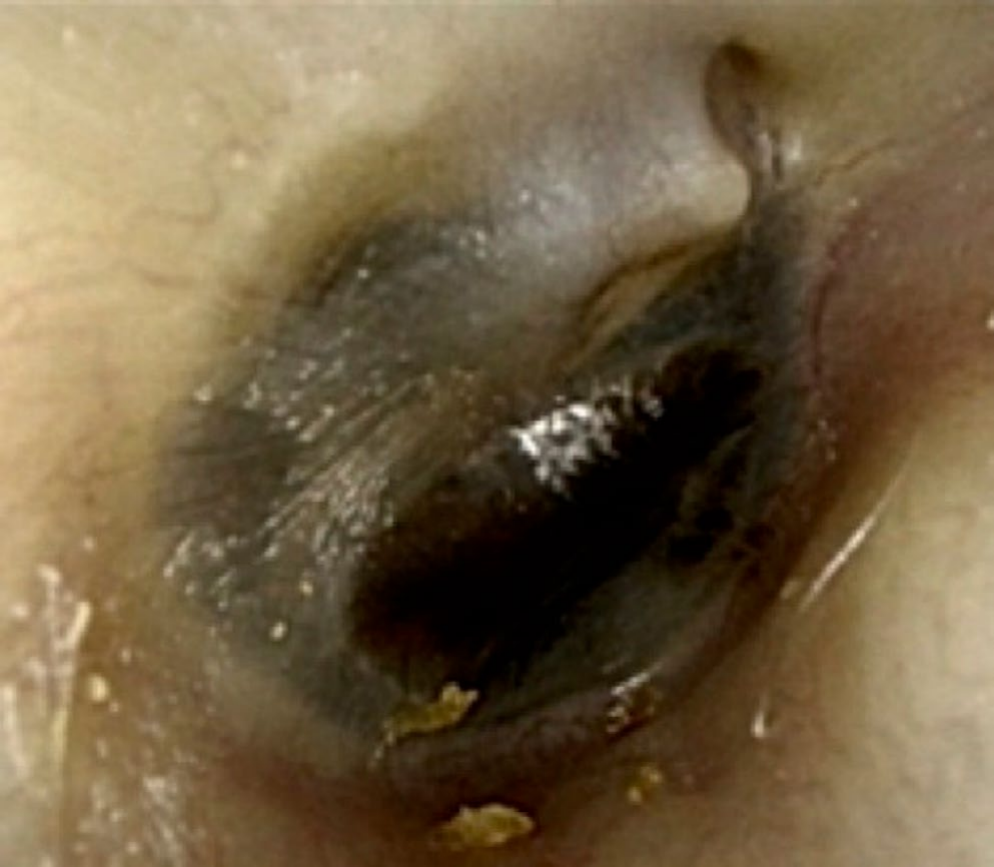
The tumor image after eight courses of nivolumab. The eardrum was visible, and the tumor was limited to the middle ear cavity

**FIGURE 5:**
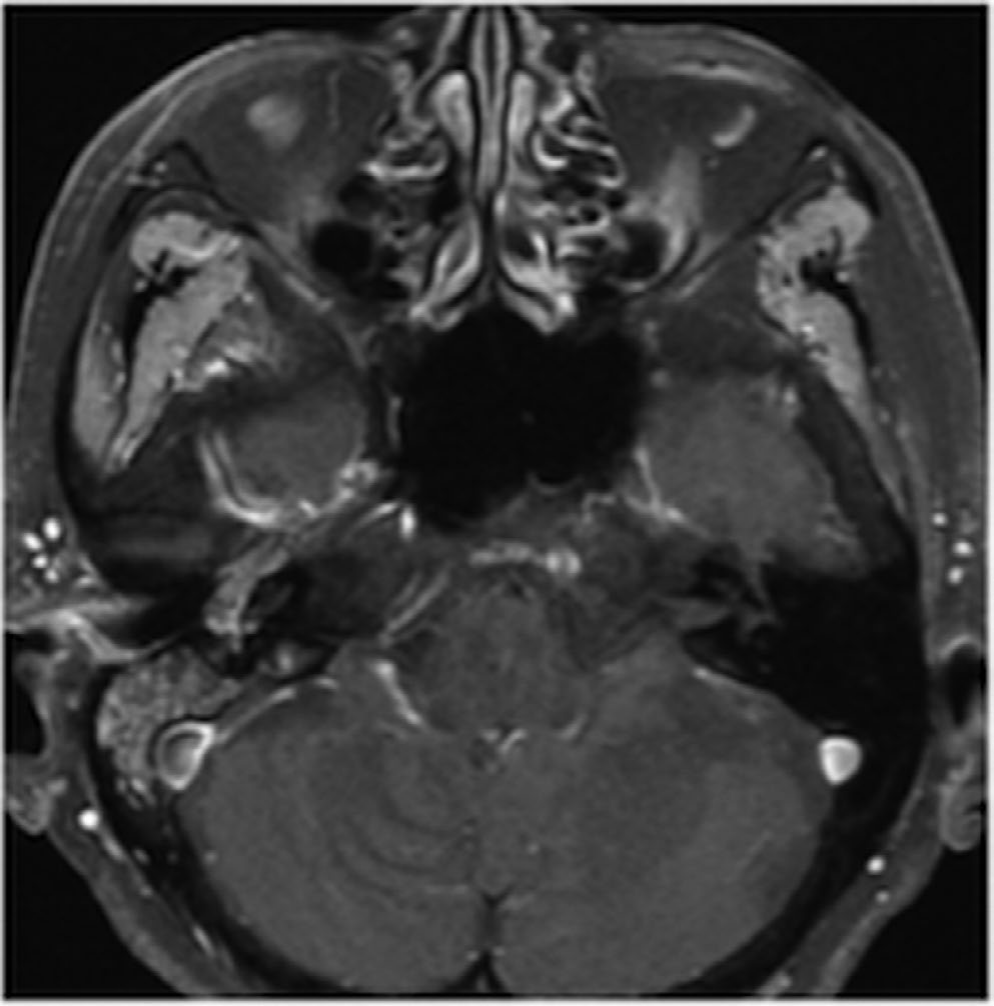
T1‐weighted axial MRI after eight courses of nivolumab. The tumor was limited to the middle ear cavity

An 80‐year‐old woman with a 2‐year history of hearing difficulty was admitted to our hospital after her primary care physician found edema of the right eardrum. Edema with active bleeding was visualized from the right eardrum to the external auditory canal (Figure 1). Computed tomography (CT) and magnetic resonance imaging (MRI) revealed a tumor that extended from the middle ear cavity to the external auditory canal (Figure 2). A fluorodeoxyglucose‐positron emission tomography (FDG‐PET) scan revealed FDG accumulation that was limited to the ear. A biopsy of the tumor revealed a mass that was comprised of melatonin‐containing large cells, confirming the diagnosis of malignant melanoma (stage T4 on the Pittsburgh staging system). Since the tumor expressed high levels of PD‐L1, the patient was treated with nivolumab (Figure 3). Three mg/kg of nivolumab was administered every 2 weeks. Reduction in tumor size was noticed immediately after treatment was begun (Figure 4). After treatment began, the patient was examined once a month and had a MRI scan every 3 months. After eight courses of treatment, the tumor was limited to the middle ear cavity and stopped bleeding (Figure 5). The patient has remained stable for more than 60 months after her first visit without serious adverse events or progression of symptoms.

## 3 DISCUSSION

The incidence rate of malignant melanomas is 1‐2 per 100 000 in Japan, among which primary mucosal melanomas account for only a few percent. Mucosal melanoma has an aggressive prognosis because of the high rate of local recurrence and distant metastasis.^14^ The overall 5‐year survival rate is no more than 30%.^15^ The treatment of malignant melanoma is surgical resection of the primary site with appropriately wide margins. However, for melanoma of the head and neck, function‐preserving resection is difficult. The effect of chemotherapy and radiation therapy on mucosal melanoma remains unknown.^16^ Recently, several reports have suggested that a PD‐1 inhibitor alone or in combination with a CTLA‐4 inhibitor is effective in treating melanoma.^16^, ^17^ However, there are few reports of the use of these immune checkpoint inhibitors in mucosal melanoma.

Of primary malignant melanomas, a tumor originating in the middle ear is rare with only 12 reports to date.^1^, ^2^, ^3^, ^4^, ^5^, ^6^, ^7^, ^8^, ^9^, ^10^, ^11^, ^12^ Early manifestations of this tumor are hearing difficulty and otorrhea. Most are misdiagnosed as serous otitis media. The treatment outcome is poor, even with extended surgical removal, and the longest survival period documented in past reports was approximately 24 months. Since our tumor was positive for PD‐L1 expression, the patient was successfully treated with nivolumab, an anti‐PD‐1 antibody.

Although direct evidence is obscure, the blockade of immune checkpoints could be a promising approach in treating primary malignant mucosal melanomas of the middle ear.

## 4 CONCLUSION

We report a case of primary mucosal malignant melanoma of the middle ear treated with a PD‐1 blockade. PD‐1 blockade is a feasible approach in treating mucosal malignant melanomas of the middle ear.

## CONFLICT OF INTEREST

None declared.

## AUTHOR CONTRIBUTIONS

HK and KT: contributed to acquisition of data, drafting the manuscript, final revision of the manuscript, and participated sufficiently in the work. SU, YH, TH, and YH: contributed to acquisition of data, drafting the manuscript, and participated sufficiently in the work.
